# Genome Sequence and Characteristics of the Microbacterium foliorum Cluster EE Bacteriophage Burgy

**DOI:** 10.1128/mra.00912-22

**Published:** 2022-10-05

**Authors:** Halle F. Van Roekel, Jacob J. Georgen, Rainy A. Kock, Sean T. Coleman

**Affiliations:** a Biology Department, Wartburg College, Waverly, Iowa, USA; Portland State University

## Abstract

Burgy is a siphovirus that was isolated from compost soil near Fremont Township, Iowa, using Microbacterium foliorum NRRL B-24224. The genome has a length of 17,453 bp and contains 25 total protein-coding genes, 20 of which were assigned functions. Based on gene content, Burgy was assigned to actinobacteriophage cluster EE.

## ANNOUNCEMENT

Bacteria of the phylum *Actinobacteria* have been identified throughout aquatic, soil, and animal microbiomes (1). Some *Actinobacteria* genera, including *Microbacterium*, can cause opportunistic infections (2, 3). The isolation and characterization of viruses that can infect *Actinobacteria*, actinobacteriophages, not only would be valuable for understanding the diversity and evolution of bacteriophages more generally but also may inform ongoing efforts to develop bacteriophages as therapeutic agents against actinobacterial infections (4). Here, we report on Burgy, a bacteriophage that infects Microbacterium foliorum.

**FIG 1:**
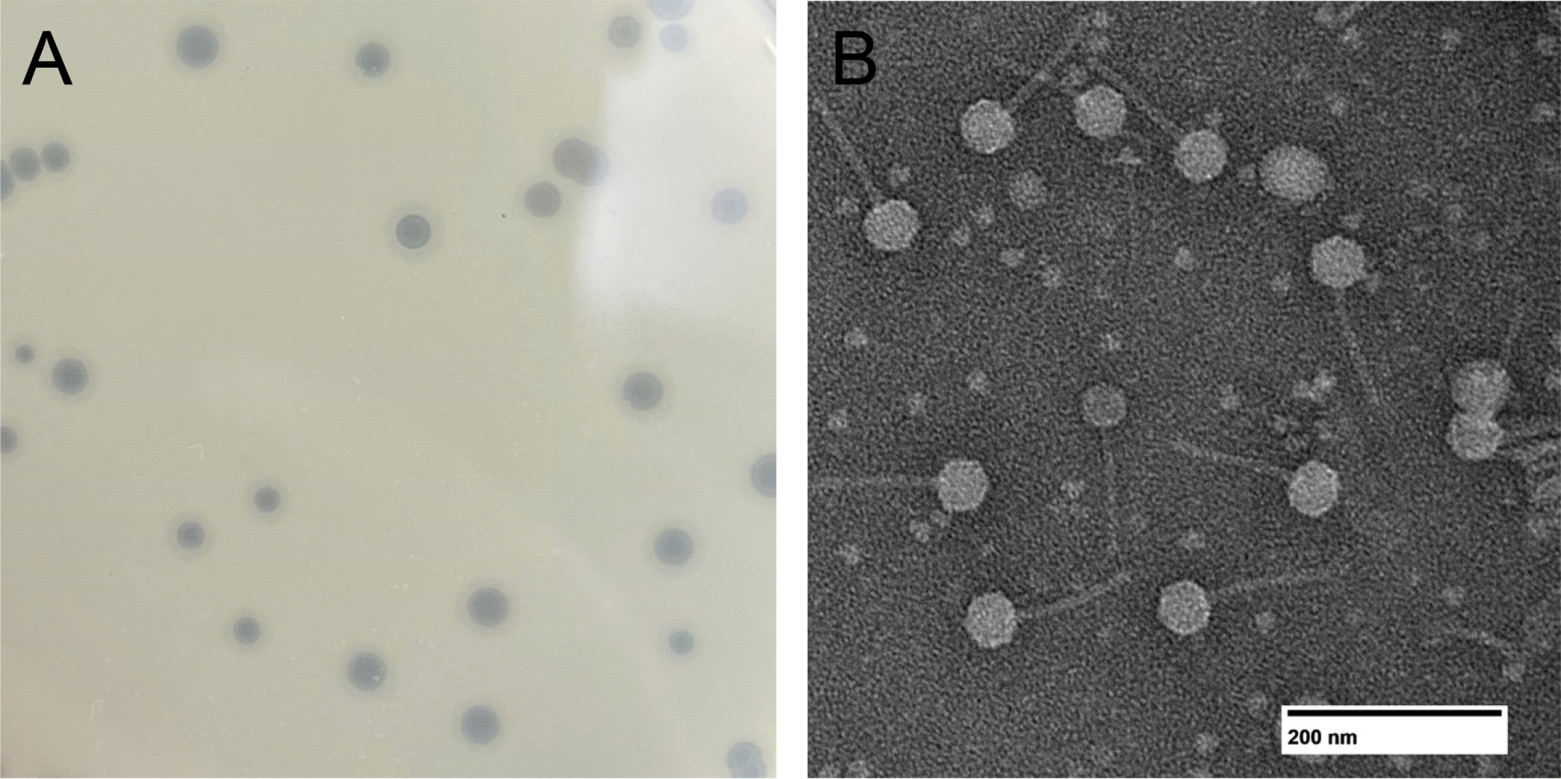
(A) Clear plaques (1 to 3 mm in diameter) formed by Burgy after 24 h of incubation at 30°C on PYCa medium with host Microbacterium foliorum NRRL B-24224. (B) Negative-staining transmission electron micrograph of Burgy, with a tail length of 113 to 119 nm and an isometric capsid 44 to 50 nm in diameter (*n* = 4).

Burgy was isolated from vermicompost soil near Fremont Township, Iowa (42.830758N, 92.621909W), using standard methods (5). Briefly, Burgy was isolated by washing the soil sample with peptone-yeast extract-calcium (PYCa) medium, filtering the wash through a 0.22-μm filter, plating the filtrate with Microbacterium foliorum NRRL B-24224 in soft agar overlay, and incubating the plate overnight at 30°C. Burgy, which forms clear plaques, was purified through three rounds of plating (Fig. 1). Transmission electron microscopy using negative staining revealed Burgy to possess *Siphoviridae* morphology, with a tail length of 113 to 119 nm and an isometric capsid 44 to 50 nm in diameter (*n* = 4) (Fig. 1).

DNA was isolated from Burgy using the Promega Wizard DNA cleanup kit. The genome was sequenced using an Illumina MiSeq sequencer (v3 reagents) after the library was prepared using the NEBNext Ultra II FS kit, yielding 640,328 single-end 150-bp reads, which constituted ~5,500-fold coverage. Raw reads were assembled and checked for completeness using Newbler v2.9 with default parameters and Consed v29, respectively, as described previously (6). The resulting genome was 17,453 bp, with 3′ single-stranded overhangs and a G+C content of 68.6%, which is similar to that of the host M. foliorum (68.7%) (7). Burgy was assigned to cluster EE based on gene content similarity of at least 35% to phages in the Actinobacteriophage Database, PhagesDB (8, 9).

The genome was initially autoannotated via Glimmer v3.02 (10) and GeneMarkS v2.5 (11) and then manually refined using Phamerator (12), DNA Master v5.23.6 (http://cobamide2.bio.pitt.edu/computer.htm), PECAAN (https://blog.kbrinsgd.org/), HHPRED (13), Starterator v1.2 (http://phages.wustl.edu/starterator), TMHMM v2.0 (14), TOPCONS v2.0 (15), and NCBI BLAST v2.13.0 (16). No tRNA genes were identified by ARAGORN v1.2.41 (17) and tRNAscan-SE v2.0 (18). All software used default parameters. The annotation process revealed 25 protein-coding genes, 20 of which could be assigned a predicted function; these included structure and assembly functions that are encoded across the first three-quarters of the genome. Within these genes, we identified a programmed translational frameshift, yielding two isoforms of the tail assembly chaperone (gp10 and gp11). Similar to other phages of cluster EE, as described previously, Burgy possesses a fusion gene encoding a capsid subunit, capsid protease, and scaffolding functions (19). The rightmost 6 genes (genes 20 to 25) encode DNA-binding proteins as well as an HNH endonuclease. All except genes 20 to 22 are transcribed rightward. Sandwiched between these DNA metabolism genes and structure and assembly genes is a gene encoding an endolysin (19).

### Data availability.

The sequencing results for Burgy are available in GenBank with accession no. ON755188 and Sequence Read Archive (SRA) accession no. SRX14443487.
